# Prognostic Value of CEA, CA19-9, CA125, CA724, and CA242 in Serum and Ascites in Pseudomyxoma Peritonei

**DOI:** 10.3389/fonc.2021.594763

**Published:** 2021-10-18

**Authors:** Lei Liang, Jingyang Fang, Xuedi Han, Xichao Zhai, Yan Song, Yiyan Lu, Qian Zhang, Ruiqing Ma

**Affiliations:** ^1^ Department of Ultrasound, Aerospace Center Hospital, Beijing, China; ^2^ Department of Myxoma, Aerospace Center Hospital, Beijing, China; ^3^ Department of Clinical Laboratory, Aerospace Center Hospital, Beijing, China; ^4^ Department of Pathology, Aerospace Center Hospital, Beijing, China; ^5^ Department of Gastroenterology, Beijing Friendship Hospital, Capital Medical University, Beijing, China

**Keywords:** Pseudomyxoma peritonei, biomarkers, ascites, serum 3/25, prognosis

## Abstract

**Purpose:**

To investigate the expression of carcinoembryonic antigen (CEA), cancer antigen 125 (CA125), CA19-9, CA724, and CA242 in serum and ascites of pseudomyxoma peritonei (PMP) patients and evaluate the predictive value of these elevated biomarkers in pathological grade, completeness of cytoreduction (CC), and survival.

**Methods:**

From May 2009 to October 2019, a total of 512 patients diagnosed with PMP through pathology in Aerospace Center Hospital were enrolled. The serum and ascites tumor biomarkers were obtained. The diagnostic values between serum and ascites biomarkers in pathology and CC were compared by the receiver operating characteristic (ROC) curves. The correlation between pathology, cytoreduction, and biomarkers was calculated by univariate and multivariate logistic regression. The associations between different numbers of elevated biomarkers and survival status were examined using univariate and multivariate backward Cox proportional hazard regression models.

**Results:**

The results showed that the areas under the ROC curves (AUROC) in the diagnosis of CC were 0.798 (95% CI: 0.760–0.836) and 0.632 (95% CI: 0.588–0.676) in serum and ascites biomarkers, respectively. The elevated serum and ascites biomarkers were independent risk factors for both pathology and CC. The 1-year, 3-year, and 5-year survival rates were 89.07%, 73.22%, and 66.94%, respectively. Longer survival was observed in patients who had less than two elevated serum biomarkers compared with those with 2–3 and 4-5 elevated serum biomarkers (p < 0.001).

**Conclusion:**

CEA, CA125, CA19-9, CA724, and CA242 in serum and ascites can be used to judge the severity and predict the resectability. Furthermore, different numbers of elevated biomarkers can help determine the prognosis of PMP.

## Introduction

Pseudomyxoma peritonei (PMP) is a syndrome characterized by mucinous effusion and mucoprotein tumor that diffusely implant in the abdominal cavity and peritoneal surface. The primary source is most often the appendix, but the source may also be colorectal, pancreatic, ovarian, or urachal. Most patients die of intestinal obstruction, perforation, malnutrition, etc., unless they undergo radical surgery (1, 2). Dr. Paul H. Sugarbaker’s cytoreductive surgery (CRS) combined with hyperthermic intraperitoneal chemotherapy (HIPEC) is associated with improved survival and is considered the current standard of treatment (3, 4). However, problems associated with incidence rate, mortality rate, and high cost make it particularly important to select patients for combined treatment (5).

The application of serum biomarkers carcinoembryonic antigen (CEA), cancer antigen 125 (CA125), CA19-9, CA724, and CA242 in colorectal, ovarian, and pancreatic cancer has been widely reported in the literature and used in the clinic (6–8). Some studies have reported the application of serum biomarkers in PMP, which play an auxiliary role in the diagnosis and prognosis of PMP. However, there are no relevant reports about ascites biomarkers in PMP (9–12). It has been reported that the immune response of biomarkers such as CEA also exists in mucus, suggesting that this kind of cell membrane glycoprotein may fall off from the surface of PMP cell membrane into mucinous ascites (13).

This study aims to investigate the expression of CEA, CA125, CA19-9, CA724, and CA242 both in serum and ascites of PMP to evaluate the correlation between abnormal expression of these biomarkers and pathological grade, completeness of cytoreduction (CC), and survival prognosis.

## Materials and Methods

### Study Population

A prospective study was designed to find the diagnostic performance of serum and ascites biomarkers. The inclusion criteria were as follows: 1) patients were treated with CRS in Aerospace Center Hospital from May 2009 to October 2019. 2) Patients were diagnosed as having PMP by surgery and pathology. 3) Ascites and serum biomarkers were routinely collected. Patients without pathological results and serum or ascites biomarkers and those who were lost to follow-up were excluded. Eleven patients without ascites were excluded because of lack of ascites, and 94 patients whose ascites cannot be obtained were excluded due to the limitation of thick mucus jelly in the peritoneal cavity. Finally, 512 patients were enrolled in the study. The study was approved by the Aerospace Center Hospital Ethics Committee with approval no. 2009QN10. All the enrolled patients signed the informed consent.

### Study Procedure and Follow-Up

Patients were hospitalized with suspected PMP diagnosis by ultrasound (US) and computed tomography (CT) in our hospital and underwent core needle biopsy or local appendectomy in other hospitals. Biomarkers in serum and ascites were examined within 2 weeks before operation. The patients received CRS combined with HIPEC. After discharge, they received biomarkers and imaging reexamination once a half year, and those who failed to come to the hospital for reexamination were followed up by telephone. The enrolled patients were followed up to February 1, 2020.

### Laboratory Tests

Here, 2–3 ml fasting venous blood and ascites were collected from all the patients, 3,000 R/min, centrifuged for 10 min, and the serum and ascites were stored at -20°C. CEA, CA125, CA19-9, CA724, and CA242 in serum and ascites of all subjects were determined by chemiluminescence method, and the automatic chemiluminescence immunity was performed by Abbott company ARCHITECT. The reagent, quantitative standard, and quality control materials used in the test are provided by the original manufacturer and operated in strict accordance with the instructions.

The critical values of biomarkers used were all referred to the range given by the manufacturer, that is, CEA >5 ng/ml, CA125 >35 U/ml, CA19-9 >30 U/ml, CA724 >12 U/ml, CA242 >20 U/ml.

### Verification of Completeness of Cytoreduction

The CC score is a score based on tumor distribution after CRS, which is considered an important prognostic indicator for patients with appendiceal mucinous tumors. No tumor or residual tumor less than 2.5 mm was scored CC 0–1, which was considered a complete CRS. The residual tumor larger than 2.5 mm was scored CC 2–3, which was considered the largest tumor removal operation (8, 9).

### Verification of Pathology

According to the World Health Organization (WHO) 2019 digestive system tumor classification standard, the patients were divided into high-grade and low-grade (14).

### Sample Size

It was calculated that at least 72 patients with 0–1 elevated serum biomarkers and 168 patients with 4–5 elevated serum biomarkers per group would be required with alpha of 0.05, beta of 0.2, and survival rates of 80% and 60%, respectively. In this cohort study, the sample size was sufficient.

### Statistical Analysis

Continuous variables were described using median [interquartile range (IQR)]. Categorical variables were described using frequency (percentage). The serum and ascites tumor biomarkers were described in both continuous and categorical variables. The receiver operating characteristic (ROC) curves and 95% confidence interval (95% CI) were used to show the differences between the number of elevated biomarkers. The optimal cutoff values were calculated with the criteria of Youden’s index. Sensitivities and specificities with 95% CI were also calculated with the optimal cutoff values. The relationship between pathology, CC, and biomarkers were calculated by univariate and multivariate logistic regression. The survival curves were plotted using the Kaplan–Meier method, and log-rank test was used for statistical analysis. The associations between biomarkers and survival status were examined using univariate and multivariate backward Cox proportional hazard regression models. All statistical tests were two-sided using a 0.05 significance level. The data analyses were performed using SAS version 9.4. The ROC curves and survival curves were drawn in MedCalc Statistical Software version 19.0.4 and GraphPad Prism version 5.0, respectively.

## Results

### Demographic Characteristics

In total, 512 patients were included in the final data analysis. The median age was 58 (IQR: 49–64.5) years old (**Table 1**). Of the study participants, 62.70% were female and 37.30% were male. Among the enrolled patients, 74.80% and 44.53% of them showed more than one abnormal level of serum and ascites biomarkers, respectively. Patients with surgical peritoneal cancer index (PCI) more than 20 accounted for 65.23% in total. The patients with CC scores of 0–1 accounted for 49.41%. There were 86.33% of the patients who received HIPEC treatment. About 95% of the tumor originally located in appendix. The pathology grades were 68.95% with low-grade and 31.05% with high-grade.

**Table 1:** The demographic characteristics of the enrolled PMP patients.

		Median (Q1–Q3)/N (%)
Age, years		58.00 (49.00–64.50)
Gender	Male	191 (37.30)
	Female	321 (62.70)
Serum CA125, U/ml	≤35	229 (44.73)
	>35	283 (55.27)
Serum CEA, ng/ml	≤5	160 (31.25)
	>5	352 (68.75)
Serum CA19-9, U/ml	≤30	268 (52.34)
	>30	244 (47.66)
Serum CA724	≤12	170 (33.20)
	>12	343 (66.80)
Serum CA242	≤20	227 (44.34)
	>20	285 (55.66)
Abnormal number of serum biomarkers	0–1	129 (25.20)
	2–3	132 (25.78)
	4–5	251 (49.02)
Ascites CA125, U/ml	≤35	281 (54.88)
	>35	231 (45.12)
Ascites CEA, ng/ml	≤5	296 (57.81)
	>5	216 (42.19)
Ascites CA19-9, U/ml	≤30	313 (61.13)
	>30	199 (38.87)
Ascites CA724	≤12	315 (61.52)
	>12	197 (38.48)
Ascites CA242	≤20	339 (66.21)
	>20	173 (33.79)
Abnormal number of ascites biomarkers	0–1	284 (55.47)
	2–3	47 (9.18)
	4–5	181 (35.35)
PCI		25.00 (13.00–32.00)
	PCI < 10	110 (21.48)
	10 ≤ PCI ≤ 20	68 (13.28)
	PCI > 20	334 (65.23)
CC scores	CC 0–1	253 (49.41)
	CC 2–3	259 (50.59)
HIPEC	Yes	442 (86.33)
	No	70 (13.67)
Origin	Appendix	486 (94.92)
	Others	26 (5.08)
Pathology	Low-grade	353 (68.95)
	High-grade	159 (31.05)

CA, cancer antigen; CC, completeness of cytoreduction; CEA, carcinoembryonic antigen; HIPEC, hyperthermic intraperitoneal chemotherapy; PCI, peritoneal cancer index; PMP, pseudomyxoma peritonei.

### The Performance of Serum and Ascites Tumor Biomarkers in the Diagnosis of Pathology Level and Completeness of Cytoreduction

**Figure 1:**
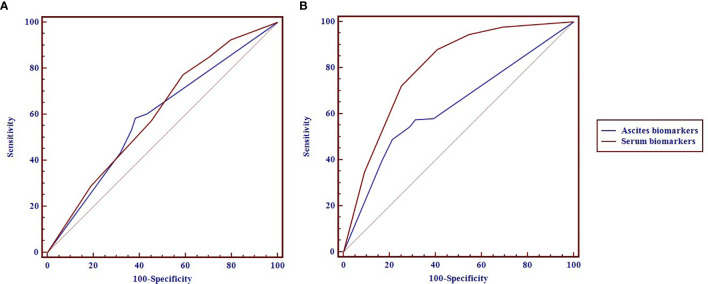
The receiver operating characteristic (ROC) curves of the serum and ascites biomarkers in diagnosis of pathology **(A)** and completeness of cytoreduction (CC) **(B)** in pseudomyxoma peritonei (PMP) patients.

As shown in **Figure 1** and **Table 2**, the areas under the ROC curves (AUROCs) in the diagnosis of pathology were 0.604 (95% CI: 0.564–0.654) and 0.587 (95% CI: 0.537–0.636) in serum and ascites biomarkers, respectively. There were no significant differences in the diagnosis of pathology between serum biomarkers and ascites biomarkers (p = 0.594). The sensitivities with optimal cutoff values were 77.4% and 58.5% in serum and ascites biomarkers, respectively. The specificities were 41.1% and 61.8%, respectively.

**Table 2:** The diagnostic performance of serum and ascites biomarkers in the prediction of pathology and completeness of cytoreduction (CC) in pseudomyxoma peritonei (PMP) patients.

	AUC (95% CI)	p-value	Sensitivity(95% CI)	Specificity(95% CI)	Cutoff value
The diagnostic performance for pathology		0.594			
Serum biomarkers	0.604 (0.564–0.654)		77.4 (70.1–83.6)	41.1 (35.9–46.4)	2
Ascites biomarkers	0.587 (0.537–0.636)		58.5 (50.4–66.2)	61.8 (56.5–66.9)	1
The diagnostic performance for CC		<0.001			
Serum biomarkers	0.798 (0.760–0.836)		88.0 (83.4–91.7)	59.3 (53.0–65.4)	2
Ascites biomarkers	0.632 (0.588–0.676)		49.0 (42.8–55.3)	78.7 (73.1–83.5)	3

The diagnostic performance of serum biomarkers for CC was better than that of ascites biomarkers (**Figure 1**). The AUROCs in the diagnosis of CC were 0.798 (0.760–0.836) and 0.632 (0.588–0.676) in serum and ascites biomarkers, respectively. In **Table 2**, the serum biomarkers were better than ascites biomarkers in the diagnosis for CC (p < 0.001). The sensitivities with optimal cutoff values were 88.0% and 49.0% in serum and ascites biomarkers, respectively. The specificities were 59.3% and 78.7%, respectively.

### The Univariate and Multivariate Logistic Regression of Biomarkers for Pathology and Completeness of Cytoreduction

In **Table 3**, the logistic regression showed that age was not correlated with pathology and CC. Male was an independent risk factor of a higher CC score (p < 0.05) but not correlated with pathology (p = 0.513 in univariate regression and p = 0.423 and p = 0.399 in multivariate regression). The elevated abnormal biomarkers of serum and ascites were independent risk factors for both pathology and CC. The results showed that the more elevated biomarkers in serum and ascites, the higher grade of pathology and higher scores of CC.

**Table 3:** The univariate and multivariate logistic regression of biomarkers for pathology and completeness of cytoreduction (CC).

	Univariate regression		Multivariate regression with serum biomarkers		Multivariate regression with ascites biomarkers	
	Odds ratio (95% CI)	p-value	Odds ratio (95% CI)	p-value	Odds ratio (95% CI)	p-value
Logistic regression for pathology						
Age, years	0.999 (0.983–1.015)	0.870	0.994 (0.978–1.011)	0.510	0.997 (0.981–1.013)	0.707
Gender, Male vs. Female	0.878 (0.595–1.297)	0.513	0.849 (0.569–1.267)	0.423	0.842 (0.565–1.255)	0.399
Serum biomarkers elevated	1.265 (1.127–1.420)	<0.001	1.270 (1.131–1.427)	<0.001	–	–
Ascites biomarkers elevated	1.161 (1.068–1.263)	0.001	–	–	1.164 (1.070–1.266)	<0.001
Logistic regression for CC						
Age, years	1.011 (0.996–1.027)	0.138	1.009 (0.990–1.028)	0.347	1.015 (0.999–1.031)	0.072
Gender, Male vs. Female	1.622 (1.130–2.329)	0.009	1.970 (1.264–3.072)	0.003	1.711 (1.166–2.512)	0.006
Serum biomarkers elevated	2.168 (1.881–2.499)	<0.001	2.185 (1.892–2.524)	<0.001	–	–
Ascites biomarkers elevated	1.286 (1.185–1.396)	<0.001	–	–	1.285 (1.183–1.396)	<0.001

### The Survival in PMP Patients of Different Elevated Biomarkers in Serum and Ascites

**Figure 2:**
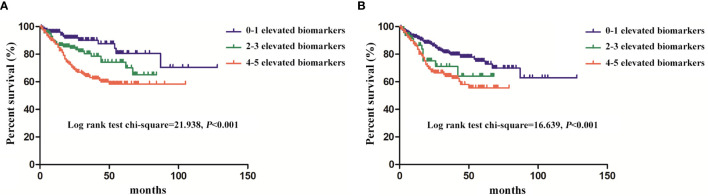
The survival of the pseudomyxoma peritonei (PMP) patients with different numbers of elevated biomarkers **(A)**, serum biomarkers; **(B)**, ascites biomarkers.

The 1-, 3-, and 5-year survival rates were 89.07%, 73.22%, and 66.94% in 512 patients, respectively. As shown in **Figure 2A**, the patients with less than two elevated serum biomarkers had a longer survival compared with patients with 2–3 and 4–5 elevated serum biomarkers (p < 0.001). The similar trends were shown in different numbers of elevated ascites biomarkers in **Figure 2B** (p < 0.001).

The univariate and multivariate Cox regression model showed that the higher level of elevated serum and ascites biomarkers, the irregular treatment of HIPEC, the high grade of pathology and PCI were independently associated with shorter survival time (**Table 4**). The elevated serum and ascites biomarkers showed a hazard ratio (HR) of 1.189 (1.024–1.379) and 1.114 (1.026–1.209), respectively.

**Table 4:** The univariate and multivariate Cox regression model of biomarkers and survival.

Cox regression model for death		Univariate regression		Multivariate regression with serum biomarkers		Multivariate regression with ascites biomarkers	
		HR (95% CI)	p-value	HR (95% CI)	p-value	HR (95% CI)	p-value
Age, years		1.008 (0.993–1.023)	0.313				
Gender, Male vs. Female		1.128 (0.792–1.606)	0.503				
Serum biomarkers elevated		1.383 (1.222–1.566)	<0.001	1.189 (1.024–1.379)	0.023		
Ascites biomarkers elevated		1.164 (1.078–1.257)	<0.001			1.114 (1.026–1.209)	0.010
Surgical PCI		1.051 (1.031–1.071)	<0.001	1.025 (1.003–1.048)	0.026	1.031 (1.011–1.051)	0.002
CC scores, 2–3 vs. 0–1	CC 0–1	2.582 (1.753–3.802)	<0.001	–	–	–	–
HIPEC, no vs. yes	Yes	3.817 (2.612–5.576)	<0.001	2.842 (1.900–4.250)	<0.001	3.132 (2.068–4.745)	<0.001
Origin, others vs. appendix		1.974 (1.035–3.767)	0.039	–	–	–	–
Pathology, high grade vs. low grade		2.985 (2.105–4.234)	<0.001	2.686 (1.882–3.833)	<0.001	2.675 (1.875–3.817)	<0.001

## Discussion

### Diagnostic Value of Preoperative Biomarkers for Pseudomyxoma Peritonei Patients

PMP develops from mucinous carcinomas that originate from appendix and/or ovary. The treatment consensus of CRS combined with HIPEC has been reached and become the first-line treatment of PMP (15).

Due to the complexity of CRS, accurate preoperative evaluation is quite important to determine the indications and surgical procedures. As a convenient tool, CEA, CA125, CA19-9, CA724, and CA242 are commonly used to monitor tumor progression or remission. In the previous literature, preoperative serum CEA, CA19-9, and CA125 concentrations were found closely related to the survival time of PMP patients, and the higher the serum biomarker concentrations were when the disease relapsed, the shorter the survival time would be after secondary tumor reduction surgery and HIPEC (10, 16, 17). However, there are different views on which biomarker to choose as the survival prediction factor of PMP in the previous literature. A few patients had false-negative serum biomarkers but suffered from persistent or recurrent diseases. We speculate that a single biomarker cannot reach an accurate prediction in PMP patients. Pathological classification and surgical resectability are also critical to the prognosis of PMP, but it has not been reported in the previous literature. This study was not aimed to study which one was the most valuable biomarker but to evaluate the relationship between the number of abnormalities and the pathological grade, CC, and survival condition of PMP from another aspect.

### Diagnostic Value of Biomarkers in Pathology

In our study, we tried to identify the pathological grade of PMP by abnormal biomarkers, which is an innovation compared with the previous studies. The results showed that the elevated abnormal biomarkers of serum and ascites were independent risk factors for pathology, the higher level of elevated biomarkers in serum and ascites, and the higher grade of pathology. In terms of cell heterogeneity in pathology, PMP was classified into high- and low-grade according to the WHO 2019 digestive system tumor classification standard (14). Compared with low-grade PMP, the more cells and even signet ring cells were observed in high-grade PMP, which means more biomarker secretion. However, cell heteroplasia does not directly affect the secretion of biomarkers. Mesothelial cells also secrete biomarkers after peritoneal stimulation. The level of biomarkers produced in this pathway is related to the area of the stimulated peritoneum.

### Diagnostic Value of Biomarkers in Deciding the Feasibility of Complete Cytoreduction

Recent studies (18, 19) indicated that preoperative imaging techniques such as CT and magnetic resonance imaging (MRI) can predict the unresectability of PMP.

The results showed that the elevated abnormal biomarkers of serum and ascites were independent risk factors for CC, the higher level of elevated biomarkers in serum and ascites, and the higher grade of CC. And the diagnostic performance of serum biomarkers for CC was better than that of ascites biomarkers.

Some biomarkers play an important role in leukocyte adhesion and extravasation (10, 20). They can promote blood-borne metastasis by mediating the adhesion between cancer cells and vascular endothelium. However, hematogenous metastasis is very rare in PMP. Therefore, we speculate that biomarkers may promote the adhesion of tumor cells to the small vessels of the peritoneum, resulting in peritoneal deposition and dissemination. It may lead to more organ involvement that the difficulty of complete surgical resection increased.

It is worth mentioning that, from this study, male was an independent risk factor of a higher CC score. In fact, the effect of gender on the overall survival (OS) of PMP patients remains to be determined. Many scholars believe that gender has no effect on prognosis, yet others think male is an independent risk factor of OS (21–25). Based on the treatment experience of our center, we do not think gender influences the prognosis of PMP but does influence the CC score. This is a novel observation; women tend to experience discomfort at an earlier stage than men due to extensive ovarian involvement, resulting in a shorter duration. In contrast, males are often diagnosed at an advanced stage due to the non-specific symptoms (26). PCI in males was higher than that in females in some special anatomical sites, such as the hepatoduodenal ligament and small intestine, which reduced the possibility of radical resection. Perhaps this observation is limited to our single center. It is indeed necessary for us to analyze the correlation between PCI in different zones and OS in a further multicenter study.

### Diagnostic Value of Biomarkers in Survival Rates

In our study, the patients with less than two elevated serum biomarkers had longer survival rates compared with patients with 2–3 and 4–5 elevated serum biomarkers. The similar trends were shown in ascites biomarkers.

Biomarkers such as CEA and CA19-9 can inhibit the differentiation of cells and promote the formation of cell adhesion, thus inhibiting apoptosis after cell abscission and enhancing metastasis ability (27–29). Some biomarkers such as CA125 are involved in the process of peritoneal metastasis by mediating the adhesion of peritoneal free tumor cells to the surface of peritoneal mesothelial cells (30–35). The elevated levels of these biomarkers are more likely to indicate peritoneal metastasis or ascites. Some biomarkers play an important role in leukocyte adhesion and extravasation. They can promote blood-borne metastasis by mediating the adhesion between cancer cells and vascular endothelium (10). Previous studies have also reported that biomarkers CEA, CA125, and CA19-9 are independent predictors of PMP survival, which was consistent with the results of our study (10, 36–38).

In the present study, high-grade pathology, high surgical PCI, and no HIPEC were also identified as independent predictors for poorer survival rates in multivariate analysis, which were consistent with the reports (39).

### Diagnostic Value of Biomarkers in Ascites for Pseudomyxoma Peritonei Patients

It has been confirmed that PMP is rarely transferred through blood; peritoneal dissemination and implantation are the main mechanisms of metastasis instead. It is the first time to study the correlation between these ascites biomarkers and pathological grade, CC, and prognosis of PMP. Nummela et al. (14) found that CEA, the glycosylphosphatidylinositol-anchored glycoprotein, was probably shed from the surface of PMP cells into the mucinous ascites. Whether biomarkers in ascites have the same tendency as serum biomarkers or not was verified. Compared with serum biomarkers, ascites biomarkers showed the same tendency to predict pathological grade and survival outcomes, while a higher correlation with CC (P<0.001). It is innovative compared with the previous studies.

### Limitations

The study was based on a prospective analysis of over 500 PMP patients from a single center database, potentially generating selection bias. Larger sample studies from multiple centers need to be included in future work.

## Conclusion

CEA, CA125, CA19-9, CA724, and CA242 in serum and ascites can be used to judge the severity, predict the resectability, and determine the prognosis of PMP, which will help oncologists to make a more comprehensive prediction and clinical treatment strategy before surgery.

## Data Availability Statement

The raw data supporting the conclusions of this article will be made available by the authors, without undue reservation.

## Ethics Statement

This study was approved by the ethics committee of the Aerospace Center Hospital. The committee’s reference number is 2009QN10. The patients/participants provided their written informed consent to participate in this study. Written informed consent was obtained from the individual(s) for the publication of any potentially identifiable images or data included in this article.

## Author Contributions

LL, RM conceived and designed the experiments; XZ, XH, and YS provided study material or patients; XZ and YL collected and assembling data; QZ, JF, XH and LL analyzed and interpreted the data; LL and QZ contributed to the draft of the manuscript; JF, XH, XZ, YS, RM and YL revised the manuscript critically for important intellectual content. All authors approved the final version of the manuscript to be submitted. All authors agreed to be accountable for all aspects of the work in ensuring that questions related to the accuracy or integrity of any part of the work are appropriately investigated and resolved. All authors contributed to the article and approved the submitted version.

## Funding

This work was supported by Gold-Bridge Funds for Beijing (grant number ZZ21054), Aerospace Center Hospital Foundation (grant number YN201710), Beijing Talents Fund (grant number 2018000021469G198), and Natural Science Foundation of Beijing Municipal (grant number 7204249).

## Conflict of Interest

The authors declare that the research was conducted in the absence of any commercial or financial relationships that could be construed as a potential conflict of interest.

## Publisher’s Note

All claims expressed in this article are solely those of the authors and do not necessarily represent those of their affiliated organizations, or those of the publisher, the editors and the reviewers. Any product that may be evaluated in this article, or claim that may be made by its manufacturer, is not guaranteed or endorsed by the publisher.

## Abbreviations

PMP, pseudomyxoma peritonei; CC, completeness of cytoreduction; CRS, cytoreductive surgery; HIPEC, hyperthermic intraperitoneal chemotherapy; CEA, carcinoembryonic antigen; CA125, cancer antigen 125; ROC, receiver operating characteristic; AUROCs, area under the ROC curves; HR, hazard ratio.

## References

[1] Carr NJ Cecil TD Mohamed F Sobin LH Sugarbaker PH González-Moreno S. A Consensus for Classification and Pathologic Reporting of Pseudomyxoma Peritonei and Associated Appendiceal Neoplasia. Am J Surg Pathol (2016) 40:14–26. doi: 10.1097/PAS.0000000000000535 26492181

[2] Qu ZB Liu LX. Management of Pseudomyxoma Peritonei. World J Gastroenterol (2006) 12:6124–7. doi: 10.3748/wjg.v12.i38.6124 PMC4088104 17036382

[3] Sugarbaker PH. Cytoreductive Surgery and Perioperative Intraperitoneal Chemotherapy as a Curative Approach to Pseudomyxoma Peritonei Syndrome. Tumori (2001) 87:S3–5. doi: 10.1177/030089160108700415 11693816

[4] Moran BJ Cecil TD. The Etiology, Clinical Presentation, and Management of Pseudomyxoma Peritonei. Surg Oncol Clin N Am (2003) 12:585–603. doi: 10.1016/S1055-3207(03)00026-7 14567019

[5] Baratti D Scivales A Balestra MR Ponzi P Di Stasi F Kusamura S. Cost Analysis of the Combined Procedure of Cytoreductive Surgery and Hyperthermic Intraperitoneal Chemotherapy (HIPEC). Eur J Surg Oncol (2010) 36:463–9. doi: 10.1016/j.ejso.2010.03.005 20363094

[6] Duffy MJ van Dalen A Haglund C Hansson L Holinski-Feder E Klapdor R. Tumour Markers in Colorectal Cancer: European Group on Tumour Markers (EGTM) Guidelines for Clinical Use. Eur J Cancer (2007) 43:1348–60. doi: 10.1016/j.ejca.2007.03.021 17512720

[7] Ning S Wei W Li J Hou B Zhong J Xie Y. Clinical Significance and Diagnostic Capacity of Serum TK1, CEA, CA 19-9 and CA 72-4 Levels in Gastric and Colorectal Cancer Patients. J Cancer (2018) 9:494–501. doi: 10.7150/jca.21562 29483954 PMC5820916

[8] Medeiros LR Rosa DD da Rosa MI Bozzetti MC. Accuracy of CA 125 in the Diagnosis of Ovarian Tumors: A Quantitative Systematic Review. Eur J Obstet Gynecol Reprod Biol (2009) 142:99–105. doi: 10.1016/j.ejogrb.2008.08.011 18995946

[9] Kozman MA Fisher OM Rebolledo BJ Valle SJ Alzahrani N Liauw W. CA 19-9 to Peritoneal Carcinomatosis Index (PCI) Ratio Is Prognostic in Patients With Epithelial Appendiceal Mucinous Neoplasms and Peritoneal Dissemination Undergoing Cytoreduction Surgery and Intraperitoneal Chemotherapy:A Retrospective Cohort Study. Eur J Surg Oncol (2017) 43:2299–307. doi: 10.1016/j.ejso.2017.09.009 28993033

[10] Kusamura S Hutanu I Baratti D Deraco M. Circulating Biomarkers: Predictors of Incomplete Cytoreduction and Powerful Determinants of Outcome in Pseudomyxoma Peritonei. J Surg Oncol (2013) 108:1–8. doi: 10.1002/jso.23329 23720095

[11] Govaerts K Lurvink RJ De Hingh IHJT van der Speeten K Villeneuve L Kusamura S. Appendiceal Tumours and Pseudomyxoma Peritonei: Literature Review With PSOGI/EURACAN Clinical Practice Guidelines for Diagnosis and Treatment. Eur J Surg Oncol (2021) 47:11–35. doi: 10.1016/j.ejso.2020.02.012 32199769

[12] Ross A Sardi A Nieroda C Merriman B Gushchin V. Clinical Utility of Elevated Biomarkers in Patients With Disseminated Appendiceal Malignancies Treated by Cytoreductive Surgery and HIPEC. Eur J Surg Oncol (2010) 36:772–6. doi: 10.1016/j.ejso.2010.05.024 20561764

[13] Nummela P Leinonen H Järvinen P Thiel A Järvinen H Lepistö A. Expression of CEA, CA19-9, CA125, and EpCAM in Pseudomyxoma Peritonei. Hum Pathol (2016) 54:47–54. doi: 10.1016/j.humpath.2016.02.022 27038681

[14] Nagtegaal ID Odze RD Klimstra D Paradis V Rugge M Schirmacher P. The 2019 WHO Classification of Tumours of the Digestive System. Histopathology (2020) 76(2):182–8. doi: 10.1111/his.13975 PMC7003895 31433515

[15] Moran B Baratti D Yan TD Kusamura S Deraco M. Consensus Statement on the Loco-Regional Treatment of Appendiceal Mucinous Neoplasms With Peritoneal Dissemination (Pseudomyxoma Peritonei). J Surg Oncol (2008) 98:277–82. doi: 10.1002/jso.21054 18726894

[16] Carmignani CP Hampton R Sugarbaker CE Chang D Sugarbaker PH. Utility of CEA and CA 19-9 Biomarkers in Diagnosis and Prognostic Assessment of Mucinous Epithelial Cancers of the Appendix. J Surg Oncol (2004) 87:162–6. doi: 10.1002/jso.20107 15334630

[17] Koh JL Liauw W Chua T Morris DL. Carbohydrate Antigen 19-9 (CA 19-9) Is an Independent Prognostic Indicator in Pseudomyxoma Peritonei Post Cytoreductive Surgery and Perioperative Intraperitoneal Chemotherapy. J Gastrointest Oncol (2013) 4:173–81. 10.3978/j.issn.2078-6891.2012.062 PMC3635178 23730513

[18] Low RN Barone RM Rousset P. Peritoneal MRI in Patients Undergoing Cytoreductive Surgery and HIPEC: History, Clinical Applications, and Implementation. Eur J Surg Oncol (2021) 47:65–74. doi: 10.1016/j.ejso.2019.02.030 30852063

[19] Sinukumar S Mehta S As R Damodaran D Ray M Zaveri S. Analysis of Clinical Outcomes of Pseudomyxoma Peritonei From Appendicular Origin Following Cytoreductive Surgery and Hyperthermic Intraperitoneal Chemotherapy-A Retrospective Study From INDEPSO. Indian J Surg Oncol (2019) 10:65–70. doi: 10.1007/s13193-018-00870-w PMC6397130 30886496

[20] Kannagi R. Molecular Mechanism for Cancer-Associated Induction of Sialyl Lewis X and Sialyl Lewis A Expression—The Warburg Effect Revisited. Glycoconj J (2004) 20:353–64. doi: 10.1023/B:GLYC.0000033631.35357.41 15229399

[21] Ansari N Chandrakumaran K Dayal S Mohamed F Cecil TD Moran BJ. Cytoreductive Surgery and Hyperthermic Intraperitoneal Chemotherapy in 1000 Patients With Perforated Appendiceal Epithelial Tumours. Eur J Surg Oncol (2016) 42:1035–41. doi: 10.1016/j.ejso.2016.03.017 27132072

[22] Chua TC Moran BJ Sugarbaker PH Levine EA Glehen O Gilly FN. Early- and Long-Term Outcome Data of Patients With Pseudomyxoma Peritonei From Appendiceal Origin Treated by a Strategy of Cytoreductive Surgery and Hyperthermic Intraperitoneal Chemotherapy. J Clin Oncol (2012) 30:2449–56. doi: 10.1200/JCO.2011.39.7166 22614976

[23] Chua TC Liauw W Morris DL. Early Recurrence of Pseudomyxoma Peritonei Following Treatment Failure of Cytoreductive Surgery and Perioperative Intraperitoneal Chemotherapy Is Indicative of a Poor Survival Outcome. Int J Colorectal Dis (2012) 27:381–9. doi: 10.1007/s00384-011-1303-8 21853235

[24] Delhorme JB Severac F Averous G Glehen O Passot G Bakrin N. Cytoreductive Surgery and Hyperthermic Intraperitoneal Chemotherapy for Pseudomyxoma Peritonei of Appendicular and Extra-Appendicular Origin. Br J Surg (2018) 105:668–76. doi: 10.1002/bjs.10716 29412465

[25] van Eden WJ Kok NFM Snaebjornsson P Jóźwiak K Woensdregt K Bottenberg PD. Factors Influencing Long-Term Survival After Cytoreductive Surgery and Hyperthermic Intraperitoneal Chemotherapy for Pseudomyxoma Peritonei Originating From Appendiceal Neoplasms. BJS Open (2019) 3:376–86. doi: 10.1002/bjs5.50134 PMC6551418 31183454

[26] Mittal R Chandramohan A Moran B. Pseudomyxoma Peritonei: Natural History and Treatment. Int J Hyperthermia (2017) 33:511–9. doi: 10.1080/02656736.2017.1310938 28540829

[27] Jin X Xu X Xu H Lv L Lu H. The Diagnostic Value of Carcinoembryonic Antigen and Squamous Cell Carcinoma Antigen in Lung Adenosquamous Carcinoma. Clin Lab (2017) . 63:801–8. doi: 10.7754/Clin.Lab.2016.160921 28397462

[28] Pakdel A Malekzadeh M Naghibalhossaini F. The Association Between Preoperative Serum CEA Concentrations and Synchronous Liver Metastasis in Colorectal Cancer Patients. Cancer Biomark (2016) 16:245–52. doi: 10.3233/CBM-150561 PMC13016458 26756614

[29] Sugarbaker PH Zamcheck N Moore FD. Assessment of Serial Carcinoembryonic Antigen (CEA) Assays in Postoperative Detection of Recurrent Colorectal Cancer. Cancer (1976) 38:2310–5. doi: 10.1002/1097-0142(197612)38:6<2310::AID-CNCR2820380618>3.0.CO;2-L 1000469

[30] Gubbels JA Belisle J Onda M Rancourt C Migneault M Ho M. Mesothelin-MUC16 Binding Is a High Affifinity, Nglycan Dependent Interaction That Facilitates Peritoneal Metastasis of Ovarian Tumours. Mol Cancer (2006) 5:50. doi: 10.1186/1476-4598-5-50 17067392 PMC1635730

[31] Seelenmeyer C Wegehingel S Lechner J Nickel W. The Cancer Antigen CA125 Represents a Novel Counter Receptor for Galectin- 1. J Cell Sci (2003) 116:1305–18. doi: 10.1242/jcs.00312 12615972

[32] Gubbels JA Felder M Horibata S Belisle JA Kapur A Holden H. MUC16 Provides Immune Protection by Inhibiting Synapse Formation Between NK and Ovarian Tumour Cells. Mol Cancer (2010) 9:11. doi: 10.1186/1476-4598-9-11 20089172 PMC2818693

[33] Epiney M Bertossa C Weil A Campana A Bischof P. CA125 Production by the Peritoneum: in-Vitro and in-Vivo Studies. Hum Reprod (2000) 15:1261–5. doi: 10.1093/humrep/15.6.1261 10831552

[34] Emoto S Ishigami H Yamashita H Yamaguchi H Kaisaki S Kitayama J. Clinical Significance of CA125 and CA72-4 in Gastric Cancer With Peritoneal Dissemination. Gastric Cancer (2012) 15:154–61. doi: 10.1007/s10120-011-0091-8 21892754

[35] Yamamoto M Baba H Toh Y Okamura T Maehara Y. Peritoneal Lavage CEA/CA125 Is a Prognostic Factor for Gastric Cancer Patients. J Cancer Res Clin Oncol (2007) 133:471–6. doi: 10.1007/s00432-006-0189-2 PMC12160893 17226046

[36] Dou H Sun G Zhang L. CA242 as a Biomarker for Pancreatic Cancer and Other Diseases. Prog Mol Biol Transl Sci (2019) 162:229–39. doi: 10.1016/bs.pmbts.2018.12.007 30905452

[37] Kim DH Oh SJ Oh CA Choi MG Noh JH Sohn TS. The Relationships Between Perioperative CEA, CA 19-9, and CA72-4 and Recurrence in Gastric Cancer Patients After Curative Radical Gastrectomy. J Surg Oncol (2011) 104:585–91. doi: 10.1002/jso.21919 21695697

[38] Li Y Guo A Tang J Wang L Wang J Yu D. Role of Preoperative Sonography in the Diagnosis and Pathologic Staging of Pseudomyxoma Peritonei. J Ultrasound Med (2013) 32:1565–70. doi: 10.7863/ultra.32.9.1565 23980216

[39] Narasimhan V Wilson K Britto M Warrier S Lynch AC Michael M. Outcomes Following Cytoreduction and HIPEC for Pseudomyxoma Peritonei: 10-Year Experience. J Gastrointest Surg (2020) 24:899–906. doi: 10.1007/s11605-019-04239-4 31090036

